# *Strongyloides*-Specific IgE Phage cDNA Clones and Development of a Novel ELISA for Strongyloidiasis

**DOI:** 10.3390/diagnostics11060985

**Published:** 2021-05-28

**Authors:** Hussain Ahmad, Norsyahida Arifin, Thomas J. Nolan, James B. Lok, Nor Suhada Anuar, Rahmah Noordin

**Affiliations:** 1 Institute for Research in Molecular Medicine (INFORMM), Universiti Sains Malaysia, Gelugor 11800, Malaysia; hussainahmad@awkum.edu.pk (H.A.); syahida_arifin@usm.my (N.A.); norsuhada@usm.my (N.S.A.); 2 Department of Microbiology, Abdul Wali Khan University, Mardan 23200, Pakistan; 3 Department of Pathobiology, School of Veterinary Medicine, University of Pennsylvania, Philadelphia, PA 19104, USA; parasit@vet.upenn.edu (T.J.N.); jlok@vet.upenn.edu (J.B.L.)

**Keywords:** *Strongyloides stercoralis*, strongyloidiasis, immunoscreening, complementary DNA (cDNA) library, recombinant antigen, serodiagnosis, IgE-enzyme linked immunosorbent assay (ELISA)

## Abstract

Strongyloidiasis, caused mainly by the nematode *Strongyloides stercoralis*, is prevalent worldwide and potentially fatal in immunosuppressed patients. We report on a new IgE biomarker to diagnose *Strongyloides* infection. Sera from two groups infected with *Strongyloides* served as positive samples: Group 1A, in which infection was confirmed by stool-microscopy and/or stool-polymerase chain reaction (PCR) and was seropositive by an IgG-enzyme linked immunosorbent assay (ELISA) and an IgG4 rapid test, and Group 1B in which infection was confirmed by stool-PCR but was seronegative. Negative samples (controls) comprised infections with other parasites (Group II) and healthy donors (Group III). Immunoscreenings of an *S. stercoralis* complementary DNA (cDNA) library were performed, and the cDNA clone with the highest diagnostic potential (clone A133) was selected for recombinant protein production and then evaluated using IgE Western blot and ELISA. The Western blot showed that the recombinant protein (rA133) was 100% reactive with Group IA (*n* = 10) and Group IB (*n* = 5), and 96% non-reactive with Groups II and III (*n* = 25). Subsequently, the IgE-ELISA was developed and showed 100% diagnostic sensitivity in Groups IA (*n* = 32) and IB (*n* = 11); and 99.3% specificity in Groups II and III (*n* = 144). In conclusion, this study has identified rA133 as a novel recombinant protein with potential diagnostic value, and that the IgE-ELISA incorporating this protein may be useful for patient diagnosis and epidemiological studies.

## 1. Introduction

Strongyloidiasis is caused by the soil-transmitted helminth *Strongyloides stercoralis* and also infrequently by *S. fuelleborni* and *S. fuelleborni kelleyi* [1,2,3]. The disease is most prevalent in tropical, subtropical, and temperate regions and is estimated to infect 370 million people globally [4,5]. The unique capacity of *S. stercoralis* for autoinfection supports lifelong infection in immunocompetent individuals and accounts for potentially fatal disseminated hyperinfection in immunosuppressed patients [6,7,8]. The risk factors for acquiring the infection are associated with skin exposure to contaminated soil containing the filariform larvae and environmental conditions that allow survival of *S. stercoralis.* The occupations at high risk for infection are farmers, gardeners, coal mine workers, and health care providers [9]. The majority of strongyloidiasis patients have uncomplicated diseases and are undiagnosed for decades. Upon immunosuppression, the infection can transform into hyperinfection syndrome and dissemination with a mortality rate of up to 85–100% [7,10].

The accurate diagnosis of *S. stercoralis* infection is essential in understanding the spread, disease burden, and clinical characterization [11]. The parasitological diagnosis by detection of larvae, either directly or via concentration and culture methods, is laborious and not sufficiently sensitive. The latter is due to the low and intermittent larval output in the feces of infected hosts. Molecular diagnosis by real-time polymerase chain reaction (PCR) provides a promising alternative; however, the need for costly reagents and specialized facilities limits it to well-equipped laboratories [11,12].

Serology is a valuable tool for the laboratory diagnosis of strongyloidiasis. Different classes of antibodies such as IgM, IgE, IgA, IgG, and its subclasses (IgG1 to IgG4) are secreted at various stages of *Strongyloides* infection. Specific IgG is the most commonly used antibody that has been used for the serodiagnosis of strongyloidiasis [13,14]. However, reports have shown that a specific IgG response was not detected in some patients with proven infection [15,16,17]. Furthermore, using IgG antibodies to diagnose *Strongyloides* infection shows cross-reactivity with filariasis and schistosomiasis [18,19]. Detection of specific IgG4 antibodies has significantly improved specificity and is useful for detecting established chronic infection [20,21]. IgM assays do not lead to clear results, and IgM levels significantly decline with time [22]. IgA is the second most prevalent antibody in serum; however, it has relatively fast catabolism and *Strongyloides* patients with severe symptoms have reduced IgA concentrations [23,24].

IgE antibodies remain for years, indicative of their origins in plasma cells or long-lived memory B cells [25]. The antibody is considered a key player in helminth infection as IgE-deficient mice are susceptible to *Schistosoma mansoni*, *Trichinella spiralis*, and *Brugia malayi* [26,27,28]. Elevated IgE levels occur in schistosomiasis, hookworm, *Trichuris*, and *Ascaris* infections in animals and humans [29,30,31,32]. Anti-*Strongyloides* IgE was detected in 90% of patients with acute infection [22,33]. A high prevalence of specific IgE is also seen in acute toxoplasmosis [34]. Treatment of strongyloidiasis with ivermectin brings about a significant decrease in levels of IgE or its disappearance within six to twelve months [35]. All of the above implies a potential role for IgE in improving the early detection of parasitic infections [34,36,37].

Furthermore, we have shown that IgE-enzyme linked immunosorbent assay (ELISA) using larval lysate was sensitive and specific in detecting strongyloidiasis [38]. For sustainable production of a diagnostic test, the use of recombinant protein is much preferred to native parasite antigens; thus, in the present study, we screened an *S. stercoralis* complementary DNA (cDNA) library with serum samples to identify cDNA clones specifically reactive to IgE antibodies in sera of *Strongyloides*-infected patients. The corresponding recombinant protein was then tested with Western blot and ELISA.

## 2. Materials and Methods

### 2.1. Serum Samples

A total of 144 serum samples were used in this study. Serum samples from anonymized patients and controls were stored frozen in the serum bank at the Institute for Research in Molecular Medicine (INFORMM), Universiti Sains Malaysia. Groups IA (*n* = 32) and IB (*n* = 11) were positive sera from *Strongyloides*-infected individuals for diagnostic sensitivity determination. Meanwhile, control sera for diagnostic specificity determination were from other infections (Groups II, *n* = 89) and healthy donors (Group III, *n* = 55). All serum samples were previously tested with a commercial IgG ELISA kit (SciMedx Corporation, Denville, NJ, USA). Additionally, Group IA and IB samples were also tested with an in-house IgG4 rapid test [39]. Group IA sera were from individuals who were positive by the IgG-ELISA and IgG4 rapid test, and their stool was positive by microscopy and/or PCR (*n* = 32). Group IB sera were positive by stool-PCR but negative by both microscopy and serologic tests (*n* = 11).

Group II sera (*n* = 89) comprise individuals with amebiasis (*n* =4), ascariasis (*n* = 4), filariasis (*n* = 11), hookworm (*n* = 8), hydatidosis (*n* = 4) schistosomiasis (*n* = 11), taeniasis (*n* = 2), toxocariasis (*n* = 19), toxoplasmosis (*n* = 5), trichuriasis (*n* = 2), giardiasis (*n* = 1), gnathostomiasis (*n* = 1), fascioliasis (*n* = 3), trichostrongylosis (*n* = 3), malaria (*n* = 5), onchocerciasis (*n* = 2), loaisis (*n* = 1), and mixed infection (ascaris/trichuris/ascaris) (*n* = 3). As stated above, Group III comprised 55 serum sample from healthy donors.

Additionally, four serum samples from individuals with high total IgE antibodies due to allergies (AbBaltis Ltd., Kent, UK) were included. The total IgE level was determined by Phadia 1000 Immunoassay Analyzer (Thermos Scientific, MI, USA). They were allergies caused by Timothy grass (grade 5), *Dermatophagoides farina* (dust mite, grade 6), silver birch tree (grade 5), and gray alder tree (grade 4). Ethical approval for using the stored serum samples at INFORMM was obtained from Universiti Sains Malaysia Human Research Ethics Committee (ref: USM/JEPeM/19070400, 4 September 2019).

### 2.2. Serum Pre-Adsorption for Immunoscreening

To reduce cross-reactivity, sera were pre-adsorbed with *Escherichia coli* antigen before performing the immunoscreening. Equal volumes of serum samples from Group IA (*n* = 5) were pooled and used as a positive sample, while sera from other parasitoses (Group II, *n* = 5) and from healthy donors in Group III (*n* = 5) were combined and used as a pooled negative sample. Individual positive and negative serum samples were also pre-adsorbed. Sera were pre-adsorbed by adding 30 µL serum to 100 mg of *E. coli* XL1-Blue pellet with 1% (*v*/*v*) thimerosal (Sigma Aldrich, St. Louis, MO, USA). The resulting combination was mixed and incubated overnight at 4 °C on a rotary shaker, then centrifuged at 10,000× *g* for 15 min at 4 °C. Serum in the supernatant was transferred to a new *E. coli* XL1-Blue pellet and incubated for 8 h on a rotary shaker at 4 °C. After another centrifugation, the serum supernatant was collected and transferred to beads coated with *E. coli* lysate. The coated beads were prepared by incubating 100 µL of microsphere beads (0.4 µm, Bangs Laboratories Inc., Fishers, IN, USA) in 250 µL *E. coli* lysate (2 mg/mL). The incubation of serum with beads was performed the same way as with *E. coli* whole-cell pellet. During each pre-adsorption stage with pellet and lysate, 5 µL was kept to assess the efficiency of the pre-adsorbed sera. The final pre-adsorbed sera were stored at −20 °C.

### 2.3. IgG-Enzyme Linked Immunosorbent Assay (ELISA) to Check the Efficiency of Serum Pre-Adsorption

An IgG-ELISA was performed to check the efficiency of serum pre-adsorption. A microtiter plate (Nunc MaxiSorp; Nalge Nunc International, Rochester, NY, USA) was coated with 5 µg/mL *E. coli* XL1-Blue antigen diluted in buffer (0.06 M carbonate buffer, pH 9.6) by overnight incubation at 4 °C. The next day, the plate was incubated for an hour at 37 °C, followed by washing five times with 200 µL/well of buffer (0.05% Tween-20 in PBS), and blocking with 200 µL/well of 3% bovine serum albumin (Amresco, Solon, OH, USA) for 1 h. The serum samples (1:100 in PBS) collected during each step of the pre-adsorption were added (duplicate wells, 100 µL/well) and incubated for 2 h at 37 °C. After a washing step, another incubation (30 min) was performed with 100 µL/well (1:4000 dilution in PBS) of mouse monoclonal anti-human IgG conjugated to horseradish peroxidase (HRP) (Invitrogen, Carlsbad, CA, USA). Finally, the plate was washed, and ABTS™ (2,2′-Azino-di-[3-ethylbenzthiazoline sulfonic acid]), as the HRP substrate, was dissolved in H_2_O_2_ in glycin/citric acid buffer (provided in kit) (Roche Diagnostics GmbH, Mannheim, Germany) and added to the wells (100 µL/well) and incubated at 37 °C for 30 min. All the incubation steps were conducted on a microplate shaker at 300 rpm, while the washing steps were performed at 700 rpm. The optical density (OD) values of the reaction mixtures were read at 405 nm/490 nm using a Thermo Multiskan Spectrum Reader (Multiskan Spectrum, Thermo Scientific, Rockford, IL, USA).

### 2.4. Immunoscreening of S. stercoralis Complementary DNA (cDNA) Library

A cDNA library was previously made from a mixture of *S. stercoralis* L3 and adult worms and constructed in λTriplEx2 vector by Clontech Laboratories Inc. (Mountain View, CA, USA) as described previously [40]. A volume of 10 µL (10^3^ phage library dilution) was added to 600 µL of *E. coli* XL1-Blue and incubated at 37 °C for 15 min to allow attachment and transduction. The mixture was added to 7 mL of melted soft top agarose, swirled, and decanted on top of the pre-warmed LB/MgSO_4_ agar plate. It was then allowed to solidify at room temperature for 10 min, followed by 10 h of incubation at 37 °C for plaque formation. Subsequently, a nitrocellulose membrane (NC) (Millipore, Bedford, MA, USA) soaked with 10 mM isopropyl β-d-1-thiogalactopyranoside (IPTG) was laid on top of the agar layers in plates containing visible plaques and incubated at 37 °C for 4 h. Spots were marked asymmetrically both on the membrane and at the edge of the plate. Next, the NC membrane was carefully removed with sterile forceps, washed three times for 5 min with Tris-buffered saline (TBS, 20 mM Tris, 150 mM NaCl) containing Tween-20 (0.05%) and blocked for 2 h with SuperBlock^TM^ (Pierce Biotechnology, Rockford, IL, USA). The membrane was then washed and incubated overnight at 4 °C with a pre-adsorbed serum sample at 1:100 dilution in TBS. The next day, the blot was washed and incubated for 2 h with monoclonal mouse anti-human IgE-HRP (Southern Biotech, Birmingham, AL, USA). Signals in the blots were then developed in the darkroom, using chemiluminescence substrate and an X-ray film (Thermo Scientific, Waltham, MA, USA). Dark spots on the film represent proteins expressed by the cDNA clones that reacted with the antibodies in the serum samples. The corresponding clones were cored out from the plate agar as plugs and added into a tube containing 200 µL lambda buffer supplemented with 5% (*v*/*v*) chloroform. The phages were allowed to diffuse into the buffer overnight at 4 °C, centrifuged for 5 min at 15,000× *g*, and the supernatant saved.

Immunoscreening was performed as described [41]. Primary immunoscreening was performed with pre-adsorbed pooled sera from strongyloidiasis patients. Reactive clones were subjected to secondary immunoscreening using pooled pre-adsorbed serum samples from *Strongyloides* patients, pooled negative sera of the individuals infected with other soil-transmitted helminths, and healthy donors’ sera. Finally, tertiary immunoscreening was performed using the individual positive and negative pre-adsorbed serum. Each NC membrane was cut into six triangular sections for incubation with six different serum samples. Percent reactivity with patient sera and non-reactivity with negative control sera were determined.

### 2.5. Post-Immunoscreening Analysis

IgE reactive cDNA clones selected for potential diagnostic value in the tertiary immunoscreening were in-vivo excised from vector λTriplEx2 and used to prepare pTriplEx2 phagemid vector. This was done by mixing 150 µL of the eluted plaque with 200 µL of *E. coli* BM25.8 overnight culture (supplemented with 100 µL of 1 M MgCl_2_). The mixture was incubated for 30 min at 31 °C without shaking, followed by the addition of 400 µL of LB broth and further incubated for 1 h at 31 °C with 225 rpm shaking. A volume of 5 µL of cell suspension was spread with a sterile glass spreader on an LB/ampicillin agar plate and incubated at 37 °C overnight for colony formation. An isolated colony was cultured, and the plasmid was purified using QIAprep Spin Miniprep Kit (QiagenGmbH, Hilden, Germany) according to the manufacturer’s instructions.

The concentration and purity of the plasmid DNA were determined using a NanoPhotometer (Implen, München, Germany), then sent to a local scientific company (First BASE Laboratories Sdn. Bhd, Malaysia) for sequencing using vector-specific primers: 5′ TriplEx2_F (5′-TCC GAG ATC TGG ACG AGC-3′) as the forward primer and 3′TriplEx2_R (5′-TAA TAC GAC TCA CTA TAG GG-3′) as the reverse primer. Sequences were analyzed using bioinformatics search tools and other public databases for nematodes.

### 2.6. Custom Cloning into a pET32a Expression System

The best clone identified in the immunoscreening and sequence analysis was selected to produce a recombinant protein. The nucleotide sequence of the selected clone was sent to a scientific company (EPOCH Life Science Inc., Missouri City, TX, USA) for codon optimization, nucleotide synthesis, and cloning into the pET32a expression system.

### 2.7. Expression and Purification of Recombinant Protein

The recombinant plasmid provided by the company was transformed into *E. coli* host cells C41 (DE3). An overnight culture of the recombinant *E. coli* (20 mL) was added to 500 mL Terrific broth (TB) supplemented with 100 µg/mL ampicillin and cultured at 37 °C with 200 rpm shaking until the OD_600_ reached 0.6. The culture was induced with 1 mM IPTG and incubated for four hours at 28 °C with 200 rpm shaking. The *E. coli* cells were collected by centrifugation (10,000× *g*, 10 min at 4 °C) using a high-speed centrifuge (Avanti J-26XPi, Beckman-Coulter, Brea, CA, USA). The cell pellet was re-suspended in cold lysis buffer (50 mM NaH_2_ PO_4_, 300 mM NaCl, 10 mM imidazole, and pH 8.0) containing protease inhibitor cocktail (Roche Diagnostics GmbH, Mannheim, Germany) at a ratio of 25:1 and 0.5 mg/mL of lysozyme (Amresco, Solon, OH, USA). After a 30-min incubation on ice, the cells were disrupted using a French Press G-M™ (Glen Mills Inc., Clifton, NJ, USA) and centrifuged at 10,000× *g* for 10 min at 4 °C. The supernatant was treated with 0.5 µg/mL DNase1 (Amresco, Solon, OH, USA), followed by incubation on ice for 30 min and centrifugation at 10,000× *g* for 30 min at 4 °C. The lysate was filtered with a 0.45 µm membrane to remove debris. The filtered lysate was incubated for 1 h with nickel-nitrilotriacetic acid (Ni-NTA) resin slurry (Roche Diagnostics GmbH, Mannheim, Germany) that was previously washed with ten column volumes (CV) of distilled water and equilibrated with 5 CV of buffer A containing 20 mM of imidazole at pH 7.4. After an hour of incubation, a gradient washing step was performed using phosphate buffers (50 mM NaH_2_PO_4_, 300 mM NaCl, and pH 7.4) containing imidazole concentrations of 20, 30, and 40 mM, and the target protein was eluted with phosphate buffer containing 250 mM imidazole. The eluted protein fractions (500 µL each) were collected and separated using sodium dodecyl sulfate-polyacrylamide gel electrophoresis (SDS-PAGE). The protein fractions with good purity were pooled and concentrated using a Vivaspin column (GE Healthcare, Buckinghamshire, UK) with a molecular weight cut-off of 10 kDa. The protein concentration was determined using Bio-Rad RC DC ^TM^ protein assay reagent (Bio-Rad, Hercules, CA, USA) and then stored at −20 °C.

### 2.8. Western Blot Analysis

The first well of a 10% SDS-PAGE gel was loaded with a protein molecular mass marker (Precision Plus Protein^TM^, Bio-Rad, Hercules, CA, USA) and the protein samples (10 µg/well) were loaded into the remaining wells. The proteins were transferred onto NC membranes (Bio-Rad, Hercules, CA, USA) by semidry blotting at 12 V constant voltages for 30 min. The NC membrane was blocked for 1 h with 5% skim milk (Nacalai Tesque, Higashitamaya-cho, Japan) diluted in TBS, followed by washing with TBS containing 0.05% Tween-20 (TBS-T), three times for 5 min each. The membrane was cut into strips, and the one with the unstained protein molecular mass marker was incubated with StrepTactin-HRP (Bio-Rad, Hercules, CA, USA) at 1:8000 (diluted in blocking solution) for 1 h at room temperature. Subsequently, it was washed three times for 5 min each with TBS-T, then kept in the same buffer until it was developed together with other strips. One strip of the NC membrane was kept in TBS-T at 4 °C and the next day incubated with anti-His-HRP (1:1000) at 4 °C for 1 h, then washed with TBS-T. It was then kept in the same buffer until it was developed together with other strips. The remaining strips were incubated overnight with serum samples (1:100 in TBS) at 4 °C. Serum samples were from *Strongyloides* patients from Groups IA (*n* = 10) and IB (*n* = 5), other parasitoses from Group II (*n* = 15), and healthy donors from Group III (*n* = 10).

The next day, the membrane strips were washed with TBS-T and incubated with monoclonal mouse anti-human IgE-HRP (Southern Biotech, Birmingham, AL, USA) at a dilution of 1:1000 in TBS for 2 h at room temperature. The reaction signals were developed in the darkroom using a chemiluminescence substrate and an X-ray film.

### 2.9. Confirmation of Protein Identity by LC-MS-MS

The purified rA133 was subjected to SDS-PAGE, and the protein band was excised and sent to the Australian Proteomic Analysis Facility (APAF) for protein identification using LC-MS-MS, i.e., Q-Exactive (Thermo Fisher Scientific, Waltham, MA, USA) mass spectrometer and Easy nLC1000 (Thermo Fisher Scientific, Waltham, MA, USA) NanoLC system.

### 2.10. Development of an IgE-ELISA Using the Recombinant Protein

Parameters for an IgE-ELISA incorporating the recombinant protein were optimized by varying the amount of recombinant protein, dilutions of primary and secondary antibodies, and the secondary antibody incubation period. Pools of three serum samples from each Group IA and IB were used as positive controls, while pools of three serum samples each from Groups II and III were used as negative controls. Additionally, a duplicate well with PBS (without serum) was used as blank.

The ELISA procedure and details were as described in Section 2.3. Briefly, a microtiter plate was coated with several concentrations of rA133 (5, 10, 20 µg/mL) diluted in carbonate overnight at 4 °C. The next day, the plate was incubated for an hour at 37 °C, followed by five times washing with 200 µL/well buffer, and blocked 1 h with 200 µL/well bovine serum albumin. Subsequently, several dilutions of serum samples (1:50, 1:100, 1:200) in PBS were incubated for 2 h at 37 °C. After another washing step, several dilutions of monoclonal mouse anti-human IgE-HRP (1:500, 1:750, 1:1000, 1:2000) in PBS were incubated for several periods (30 min, 1 h, 2 h). Finally, the plate was washed, and the ABTS substrate (100 µL/well) was incubated at 37 °C for 30 min. The OD values of the reaction mixtures were read at 405 nm/490 nm using an ELISA reader.

The optimized parameters were used to determine the diagnostic sensitivity of the IgE-ELISA with sera from Groups IA (*n* = 32) and IB (*n* = 11). Diagnostic specificity was evaluated using Groups II (*n* = 89) and III (*n* = 55). The cut-off value (COV) was determined via the receiver operator characteristic curve (ROC) analysis.

### 2.11. Statistical Analysis

The diagnostic sensitivity of the IgE-ELISA was determined as the number of positive sera with ODs greater than the COV divided by the total number of sera in Groups IA and IB. The diagnostic specificity of the IgE-ELISA was determined as the number of negative sera with OD of less than the COV divided by the total number of sera in Groups II and III. The normality of the data was checked by Shapiro–Wilk test and Kolmogorov-Smirnov test, then a one-way ANOVA followed by Kruskal–Wallis test was performed using Graph Pad Prism version 8.0.2 (GraphPad Software, San Diego, CA, USA) to determine the *p*-value. A *p*-value of less than 0.05 was considered statistically significant. ROC analysis of the IgE-ELISA results was performed using MedCalc statistical software 19.0.7.

## 3. Results

### 3.1. Serum Pre-Adsorption

The OD_405_ readings of IgG-ELISA of Group IA, Group II, and Group III before and after pre-adsorption with E. *coli* XL1-Blue pellet and lysate are shown in Figure S1 in Supplementary Materials. The progressive decrease in OD_405_ values with each round of serum pre-adsorption was observed, indicating a depletion of *E. coli* reactive antibodies in the serum. An OD value of less than 0.1 was considered as a well pre-adsorbed serum suitable for immunoscreening. A serum sample of OD_405_ more than 0.1 was subjected to another round of serum pre-adsorption.

### 3.2. Immunoscreening of S. stercoralis cDNA Library

Eleven rounds of primary immunoscreening were performed with four plates in each round, and each plate containing approximately 250–300 clones. Approximately 11,000 phage clones were screened with a pooled positive serum of Group IA, which led to the identification of 122 IgE-reactive cDNA clones. They were cored out from the plates and subjected to secondary immunoscreening. A total of 27 out of the 122 clones that were only reactive to pooled positive serum and were non-reactive to pooled negative serum were selected for further screening. The tertiary immunoscreening was performed with individual serum samples, and the results showed that six clones had 87.5–100% reactivity with positive sera and 80–92% non-reactivity with negative sera (Table 1). Clone A133 was reactive with all (100%) positive sera (*n* = 10) and non-reactive with 92% of negative sera (*n* = 25). Figure S2 in Supplementary Materials shows some images of the reactivity/non-reactivity of clone A133.

### 3.3. Sequence Analysis

The DNA inserts of the final six clones were in-vivo excised, sequenced, and analyzed. The results are shown in Table 2. All clones had 100% nucleotide sequence identity with *S. stercoralis*, while the amino acid sequence identity ranged from 84–100%. Clone A133 and 3A.1 showed the same sequence identity, which corresponded to their similar immunoscreening results (Table 2). The sequence of clone A133 revealed a complete open reading frame of 723 nucleotides containing start and stop codons that encoded 240 amino acids. It showed 100% similarity with *S. stercoralis* genome assembly *S_stercoralis*_PV0001, scaffold SSTP_contig0000002 Sequence ID: LL999051.1 (identities = 723/723 [100%], gaps = 0/723 [0%]). The translated protein analysis of A133 revealed the highest similarity to *S. ratti* PDZ signaling domain protein (GH21964p) (identities = 207/237 [87%], positives = 225/237 [94%], and gaps = 0/237 [0%]) Sequence ID: CEF67580.1.

### 3.4. SDS-PAGE and Western Blot

**Figure 1:**
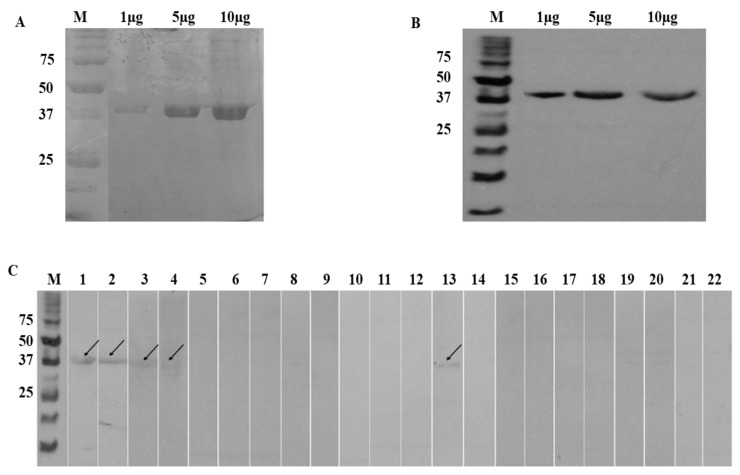
(**A**) SDS-PAGE analysis of 1, 5, and 10 µg of pooled rA133 from the eluted fractions after purification. The ~40 kDa protein band corresponds to rA133; (**B**) Western blot analysis of 1, 5, and 10 µg of purified His-tagged rA133 using Anti-His HRP as a probe. The 40 kDa protein band corresponds to r A133; (**C**) IgE Western blot analysis of rA133. The arrows show the rA133 protein bands on the nitrocelulose strips. Strip M is the unstained protein marker (Bio-Rad). Positive samples (strongyloidiasis sera) were tested as follows: Group IA serum (strips 1–2) and Group IB (strips 3–4). Group II control (other infections) serum samples were tested as follows: ascariasis (strips 5–7); filariasis (strips 8–10); hookworm infection (strips 11–13); toxocariasis (strips 14–16); schistosomiasis (strips 17–18); giardiasis (lane 19). Group III healthy individuals were tested with strips 20–22. A 40 kDa protein band that corresponded to rA133 was observed in strips 1–4. Strip 13 tested with serum from hookworm infection showed a false-positive result.

The custom cloned rA133 was expressed in *E. coli* and purified using affinity chromatography. Figure 1A shows the SDS-PAGE analysis of the affinity-purified fractions of the rA133, and Figure 1B showed the results of the anti-His Western blot that detected the rA133 via its histidine tag.

The optimal conditions obtained for IgE-Western blot that distinguished between positive and negative serum samples were 10 µg of antigen concentration, 1:100 primary antibody dilution, and 1:1000 of secondary antibody dilution. Western blot was performed using 15 positive serum samples from strongyloidiasis patients (Group IA, *n* = 10 and Group IB, *n* = 5); and 25 negative serum samples from patients infected with other parasitoses and healthy samples (Group II, *n* = 15 and Group III; *n* = 10 respectively). The results (Figure 1C) revealed that a band of 40 kDa, which matched the size of the recombinant A133 protein, was found with all 15 positive serum samples provided (100% reactivity) and was 96% non-reactive when tested with control serum sample (*n* = 25). The one serum that was cross-reactive in rA133 IgE-Western blot was a hookworm serum.

### 3.5. LC-MS-MS

The LC-MS-MS analysis was performed to validate the identity of rA133. The LC-MS-MS report showed that the protein was “PDZ domain-containing protein OS = *Strongyloides stercoralis* OX = 6248 PE = 4 SV = 1” and identified 13 unique peptides with sequence coverage of 41%.

### 3.6. IgE-ELISA Using rA133

**Figure 2:**
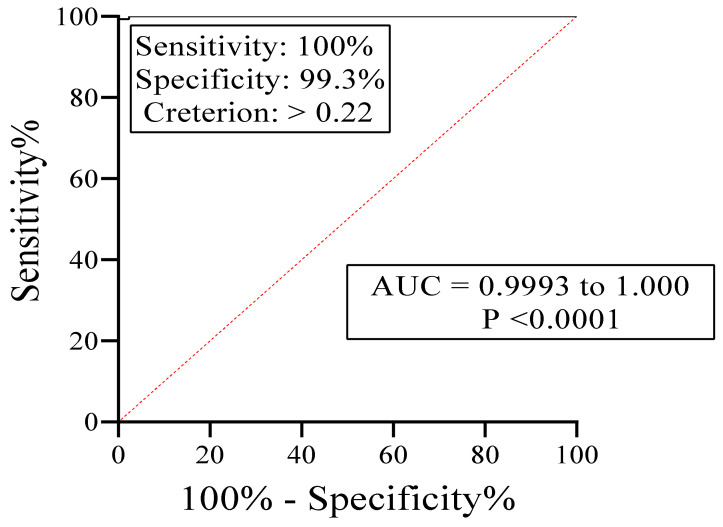
Receiver operating characteristic (ROC) analysis of the IgE-ELISA using rA133. The cut-off value (COV) is 0.22 and the area under the ROC curve (AUC) is 0.9998 (95% confidence interval = 0.9993 to 1.000).

The optimized parameters obtained for rA133 IgE-ELISA that best differentiate positive and negative controls sera were as follows: 10 µg/mL of rA133 on the plate wells, 1:100 of serum dilution in PBS, and the 1:750 dilution in PBS of IgE-HRP with 2-h incubation. The *Strongyloides*-specific IgE responses by ELISA showed a cut-off optical density (OD) value (COV) of 0.22 and the area under the “receiver operator characteristic” (ROC) curve of 0.9998 (Figure 2).

Diagnostic sensitivity of 100% was achieved with 43 sera of Groups IA and IB. The diagnostic specificity was 99.3% with 144 sera of Groups II and III. In addition, the high total IgE serum samples from patients with four different allergies were not reactive (Table 3).

**Figure 3:**
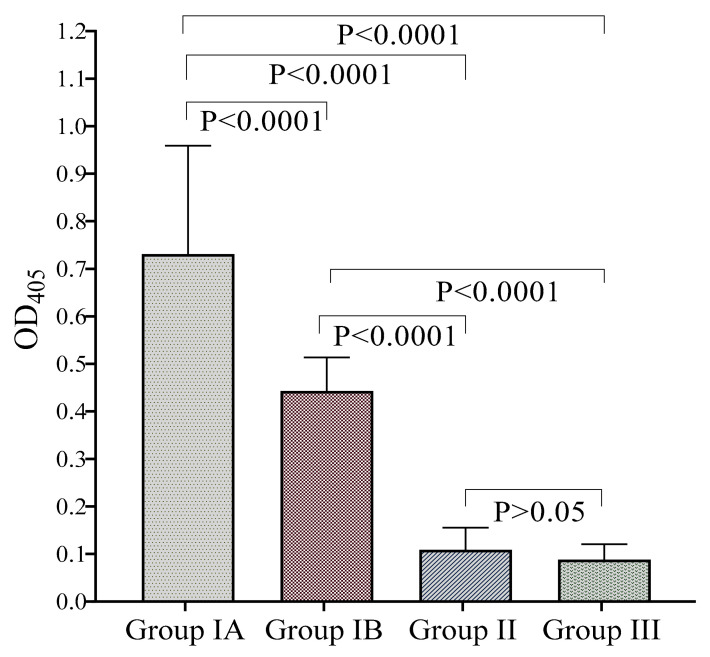
One-way ANOVA with Bonferroni multiple comparison test of the optical density (OD405) values (mean + 1SD) of all serum groups. All the groups have significant *p*-value < 0.0001 except for Groups II and III, which have statistically nonsignificant *p*-value > 0.05.

**Figure 4:**
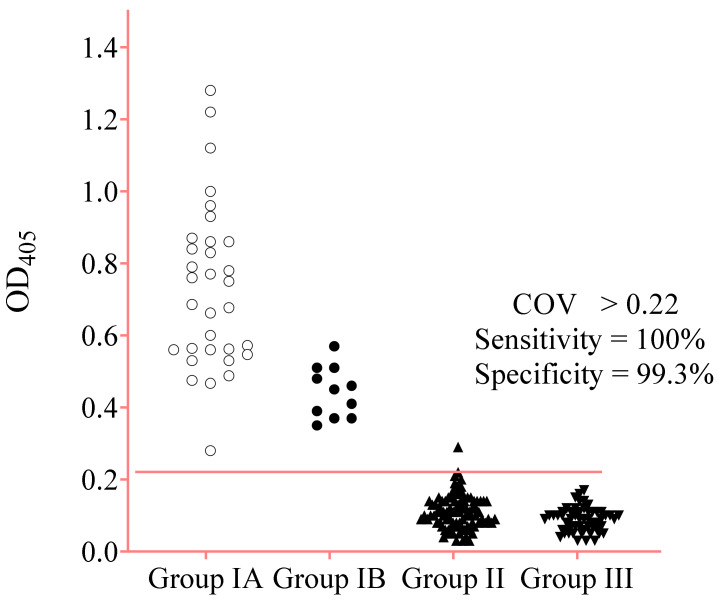
The distribution of optical density (OD_405_) values of different serum groups by IgE-ELISA. The cut-off value OD_405_ value (COV) to discriminate between positive and negative results was 0.22.

In Figure 3, one-way ANOVA shows that the average OD_450_ values between the positive samples (Group IA and IB) and negative samples (Group II and III) were significantly different (*p* < 0.0001). The Bonferroni multiple comparison test between the two positive sera groups showed significantly higher average OD values (*p* < 0.0001) of Group IA than IB. Meanwhile, the average ODs between Groups II and III were not significantly different (*p* > 0.05). The distribution of OD_450_ values of the positive and negative serum samples by IgE-ELISA is shown in Figure 4.

## 4. Discussion

*Strongyloides*-infections are commonly asymptomatic, while mildly symptomatic patients usually present with uncharacterized symptoms that mimic infections by other helminths [42]. A severe and potentially fatal infection can occur in immunosuppressed patients, with autoinfection progressing to disseminated hyperinfection. Early laboratory diagnosis can help prevent these severe consequences of *S. stercoralis* infection.

In the present study, a *Strongyloides* cDNA library was screened with serum samples to identify reactive clones specific to IgE antibodies. The serum samples were first depleted of antibodies reactive to *E. coli*. Recombinant clone A133 demonstrated 100% reactivity with positive sera (*n* = 10) and 92% non-reactivity with control sera (*n* = 25). The deduced amino acid sequence of clone A133 revealed identity to the PDZ domain (GH21964p). The name PDZ is derived from the first letter of three proteins in which this domain was observed, i.e., 95 kDa postsynaptic density protein (PSD 95), disc large tumor suppressor in *Drosophila* (DlgA), and a protein called zonula occludens-1 (Zo-1) [43]. This domain is also referred to as the DHR domain (Discs-large homologous regions) or GLGF motif (due to a highly conserved four residue GLGF sequence within the domain) [44]. The PDZ domain functions as a protein-protein interaction module and recognizes a specific C-terminal motif in the partner protein [45]. The interaction of the PDZ domain in one protein and the PDZ motif in the partner protein leads to the creation of protein scaffolds and signaling networks. Therefore, the PDZ domain is considered a novel target in drug discovery [46]. It is also among the important modules reserved in the parasite to mediate multiple biological processes. The PDZ domain protein from *Schistosoma japonicum* (SjGIPC3) was up-regulated after the invasion of the host, suggesting its role in parasite-host interaction [47]. Although the PDZ domains are conserved in various organisms, detailed information on the *Strongyloides*-derived PDZ domain has not been reported.

A133 was custom cloned into the pET32a vector system, and the recombinant protein (rA133) was expressed as a dual tag protein, i.e., thioredoxin and histidine. LC-MS-MS confirmed that the 40 kDa band matched PDZ domain-containing protein OS = *Strongyloides stercoralis* OX = 6248 PE = 4 SV = 1. An initial assessment of the immunoreactivity of rA133 was performed using an IgE-Western blot with Group IA (*n* = 10) and Group IB (*n* = 5) patients’ samples. Since Group IB samples were negative by IgG-ELISA and IgG4 rapid test, they were presumed as likely to be from patients with acute infection, while Group IA samples were likely to be from patients with chronic infection. The IgE-Western blot detected both cases of probable acute and chronic cases of strongyloidiasis with 100% sensitivity. The specificity of 96% (*n* = 25) was determined with 15 negative serum samples from patients infected with other diseases (Group II) and 10 serum samples from healthy individuals (Group III). One of the hookworm serum samples was false positive with the IgE-Western blot. Subsequently an IgE-ELISA was developed using rA133, and it showed 100% sensitivity (*n* = 43) and 99.3% specificity (*n* = 144). The serum sample that cross-reacted in the IgE-ELISA was the same hookworm serum that cross-reacted in the IgE-Western blot. The serum sample could be from a patient who is co-infected with hookworm and *Strongyloides*, or recently cured of the infection. Nevertheless, to confirm that there is no true cross-reaction with antibodies to hookworm, future evaluations of the IgE-ELISA should include a much bigger number of sera from hookworm infected individuals.

The IgE-ELISA OD_405_ values of sera from Groups IA and IB were mostly low, and only four Group IA serum samples showed OD_405_ values greater than 1.0. Nevertheless, ANOVA on the IgE-ELISA results showed a significant difference (*p*-value < 0.0001) between patients’ sera (Groups IA and IB) and negative control sera (Groups II and III). Thus, the IgE-ELISA using rA133 can differentiate *Strongyloides* patients from individuals who are healthy and/or have other infections. The Bonferroni multiple comparison test in one-way ANOVA among all the groups showed statistically significant results, except for Groups II versus III (both negative control samples). The ability of the IgE-ELISA to detect Group IB samples that were undetectable by the IgG and IgG4 assays is notable. Also, there was a significant difference (*p* < 0.0001) between average OD_450_ values of Groups 1A and 1B. Since the sample size was small for both groups, further studies are needed to ascertain whether the IgE-ELISA can differentiate patients with probable early and chronic infections.

In our earlier report on IgE-ELISA using sonicated *Strongyloides* larval crude (unpurified) lysate antigen, the range of OD_405_ values obtained with Group IA sera was 0.231 to 0.680 (mean 0.432), and Group IB ranged from 0.227 to 0.429 (mean 0.297) [38]. In the present study, the IgE-ELISA OD_405_ values of Group IA ranged from 0.280 to 1.220 (mean 0.730), and Group IB ranged from 0.351 to 0.573 (mean 0.442). In general, the OD_405_ values of IgE-ELISA using the recombinant A133 antigen were higher than those obtained using the native parasite antigen.

Overall, this study showed that rA133 could be a good diagnostic marker for *Strongyloides* infection. The initial evaluation results show it has good potential for further studies to confirm its diagnostic value in detecting both acute and chronic cases of strongyloidiasis. The sensitivity and specificity findings in this study are consistent with those observed in our earlier IgE-ELISA using larval lysate [38]. The results are also consistent with other researchers’ reports that IgE is an important marker for strongyloidiasis, especially in cases of probable early/acute infection [33,48]. Previously an IgE-ELISA using recombinant strongylastacin antigen demonstrated 93% sensitivity (*n* = 15) and 100% specificity (*n* = 15) [49]. Thus, our study showed higher sensitivity but similar specificity, although the cross-reactivity assessment of recombinant strongylastacin-based assay used only 15 sera from filariasis patients and healthy individuals. Recombinant cDNA clones 5a and 12 were also utilized in a *Strongyloides* IgE-ELISA. No cross-reactions were seen with control sera, which included filariasis patients. However, Western blot did not detect parasite-specific IgE antibodies in 3 out of 12 patients with chronic *Strongyloides* infection [50].

## 5. Conclusions

The present study has identified a novel recombinant protein with potential diagnostic value. The IgE-ELISA developed may be able to replace IgG-ELISA for laboratory diagnosis of suspected patients. The ELISA detected early infections, which may be missed due to undetectable IgG and IgG4 antibodies. When used in epidemiological studies, the IgE-ELISA may provide a more accurate prevalence data since asymptomatic people with early infection can be identified. Further studies to validate the diagnostic potential of the IgE-ELISA using rA133 are thus warranted.

## 6. Patents

The patent for the use of rA133 for the diagnosis of strongyloidiasis has been filed in Malaysia (PI 2020000313) and PCT (PCT/MY2020/050044).

## Figures and Tables

**Table 1:** Evaluation results of reactivity/non-reactivity of selected complementary DNA (cDNA) clones after tertiary immunoscreening.

Evaluation	IgE Detected Clones					
	A132	A133	FB	A51	A31	3A.1
Reactivity with positive serum (%), *n* = 10	100	100	87.5	100	100	100
Non-reactivity with negative serum (%), *n* = 25	86	92	80	82	86	91

**Table 2:** Sequence analysis of the final six IgE-reactive cDNA clones.

Nucleotide Sequence Analysis				Amino Acid Sequence Analysis		
IgE Detective Clone	Gene Bank Accession Number	BLAST Result	Identity to *Strongyloides* sp. Sequence	Gene Bank Accession Number	BLAST Result	Identity to *Strongyloides* sp. Sequence
A132	LL999048.1	*Strongyloides stercoralis* genome assembly, *S._stercoralis*_PV0001, scaffold SSTP_scaffold0000001	100%	CEF67406.1	*Strongyloides ratti* Oligosaccharyl transferase complex subunit (OSTC)	96%
A133	LL999051.1	*Strongyloides stercoralis* genome assembly *S._stercoralis*_PV0001, scaffold SSTP_contig0000002	100%	CEF67580.1	*Strongyloides ratti* PDZ signaling domain protein (GH21964p)	87%
FB	LL999088.1	*Strongyloides stercoralis* genome assembly, *S._stercoralis_*PV0001, scaffold SSTP_contig0000026	100%	CEF61968.1	*Strongyloides ratti* 40S ribosomal protein S5	100%
A51	LL999049.1	*Strongyloides stercoralis* genome assembly *S._stercoralis*_PV0001, scaffold SSTP_scaffold0000002	100%	-	No significant similarities found	-
A31	LL999050.1	*Strongyloides stercoralis* genome assembly *S._stercoralis*_PV0001, scaffold SSTP_contig0000001	100%	CEF64458.1	*Strongyloides ratti,* MSP domain and PapD-like domain-containing protein	97%
3A.1	LL999051.1	*Strongyloides stercoralis* genome assembly *S._stercoralis_*PV0001, scaffold SSTP_contig0000002	100%	CEF67580.1	*Strongyloides ratti* PDZ signaling domain protein (GH21964p)	84%

**Table 3:** Summary of evaluation of rA133 IgE-ELISA tested with different groups of serum samples.

Samples	*N*	Reactivity		Sensitivity	Specificity
		Positive	Negative		
Strongyloidiasis Group IA and Group IB	43	43	0	100%	-
Group IA	32	32	0	-	-
Group IB	11	11	0	-	-
Negative Control Group II and Group III	144	1	143		99.3%
Other infections (Group 11)				-	
Amoebiasis	4	0	4		
Ascariasis	4	0	4	-	-
Brµgian filariasis	7	0	7	-	-
Bancroftian filariasis	4	0	4	-	-
Hookworm infection	8	1	8	-	-
Hydatidosis	4	0	4	-	-
Schistosomiasis	11	0	11	-	-
Taeniasis	2	0	2	-	-
Toxocariasis	19	0	19	-	-
Toxoplasmosis	5	0	5	-	-
Trichuriasis	2	0	2	-	-
Giardiasis	1		1		
Gnathostomiasis	1		1		
Fascioliasis	3		3		
Trichostrongylosis	3		3		
Malaria	5		5		
Onchocerciasis	2		2		
Loaisis	1		1		
Mixed infection (ascaris/trichuris/hookworm)	3	0	3		
Healthy donors (Group III)	55	0	55	-	-
Serum samples from patients with high IgE due to allergies (excluded from diagnostic specificity determination)	4	0	4		

## Data Availability

The raw data of this study can be made available upon request.

## Supplementary Materials

The following are available online at https://www.mdpi.com/article/10.3390/diagnostics11060985/s1, Figure S1: OD405 readings of Group IA, Group II, and Group III serum samples before pre-adsorption and after pre-adsorption processes with whole-cell and lysate of E. coli; Figure S2: A. Reactivity with positive sera B. non-reactivity with negative sera of clone A133 us-ing pre-adsorbed individual serum samples and probed with IgE -HRP.

## References

[1] Schad G.A., Grove D.I. (1989). Morphology and life history of *Strongyloides stercoralis*. Strongyloidiasis a Major Roundworm Infection of Man.

[2] Thanchomnang T., Intapan P.M., Sanpool O., Rodpai R., Tourtip S., Yahom S., Kullawat J., Radomyos P., Thammasiri C., Maleewong W. (2017). First molecular identification and genetic diversity of *Strongyloides stercoralis* and *Strongyloides fuelleborni* in human communities having contact with long-tailed macaques in Thailand. Parasitol. Res..

[3] Sheild J.M., Kow F., Shield J.M., Kow F. (2013). A comparative study of intestinal helminths in pre-school age urban and rural children in Morobe Province, Papua New Guinea. Papua New Guinea Med. J..

[4] Bisoffi Z., Buonfrate D., Montresor A., Requena-Méndez A., Munoz J., Krolewiecki A.J., Gotuzzo E., Mena M.A., Chiodini P.L., Anselmi M. (2013). *Strongyloides stercoralis*: A plea for action. PLoS Negl. Trop. Dis..

[5] Schar F., Trostdorf U., Giardina F., Khieu V., Muth S., Marti H., Vounatsou P., Odermatt P. (2013). *Strongyloides stercoralis*: Global Distribution and Risk Factors. PLoS Negl. Trop. Dis..

[6] Utzinger J., Hatz C., Adjossan L., Sieto B., Silué K.D., Becker S.L., Kern W.V., Koné S., N’Goran E.K. (2011). Diagnosis, Clinical Features, and Self-Reported Morbidity of *Strongyloides stercoralis* and Hookworm Infection in a Co-Endemic Setting. PLoS Negl. Trop. Dis..

[7] Mejia R., Nutman T.B. (2012). Screening, prevention, and treatment for hyperinfection syndrome and disseminated infections caused by *Strongyloides stercoralis*. Curr. Opin. Infect. Dis..

[8] Keiser P.B., Nutman T.B. (2004). *Strongyloides stercoralis* in the Immunocompromised Population. Clin. Microbiol. Rev..

[9] Puthiyakunnon S., Boddu S., Li Y., Zhou X., Wang C., Li J., Chen X. (2014). Strongyloidiasis—An insight into its global prevalence and management. PLoS Negl. Trop. Dis..

[10] Lam C.S., Tong M.K.H., Chan K.M., Siu Y.P. (2006). Disseminated strongyloidiasis: A retrospective study of clinical course and outcome. Eur. J. Clin. Microbiol. Infect. Dis..

[11] Buonfrate D., Sequi M., Mejia R., Cimino R.O., Krolewiecki A.J., Albonico M., Degani M., Tais S., Angheben A., Requena-Mendez A. (2015). Accuracy of five serologic tests for the follow up of *Strongyloides stercoralis* infection. PLoS Negl. Trop. Dis..

[12] Ruiz E.F., Terashima A., Pineda-Reyes J., Vasquez-Rios G., Marin R., Pineda-Reyes R. (2019). *Strongyloides stercoralis* hyperinfection syndrome: A deeper understanding of a neglected disease. J. Parasit. Dis..

[13] Schaffel R., Nucci M., Carvalho E., Braga M., Almeida L., Portugal R., Pulcheri W. (2001). The value of an immunoenzymatic test (enzyme-linked immunosorbent assay) for the diagnosis of strongyloidiasis in patients immunosuppressed by hematologic malignancies. Am. J. Trop. Med. Hyg..

[14] Genta R.M., Schad G.A., Hellman M.E. (1986). *Strongyloides stercoralis*: Parasitological, immunological and pathological observations in immunosuppressed dogs. Trans. R. Soc. Trop. Med. Hyg..

[15] Ming D.K., Armstrong M., Lowe P., Chiodini P.L., Doherty J.F., Whitty C.J.M., McGregor A.C. (2019). Clinical and Diagnostic Features of 413 Patients Treated for Imported Strongyloidiasis at the Hospital for Tropical Diseases, London. Am. J. Trop. Med. Hyg..

[16] Bisoffi Z., Buonfrate D., Sequi M., Mejia R., Cimino R.O., Krolewiecki A.J., Albonico M., Gobbo M., Bonafini S., Angheben A. (2014). Diagnostic Accuracy of Five Serologic Tests for *Strongyloides stercoralis* Infection. PLoS Negl. Trop. Dis..

[17] Loutfy M.R., Wilson M., Keystone J.S., Kain K.C. (2002). Serology and eosinophil count in the diagnosis and management of strongyloidiasis in a non-endemic area. Am. J. Trop. Med. Hyg..

[18] Conway D.J., Atkins N.S., Lillywhite J.E., Bailey J.W., Robinson R.D., Lindo J.F., Bundy D.A.P., Bianco A.E. (1993). Immunodiagnosis of *Strongyloides stercoralis* infection: A method for increasing the specificity of the indirect ELISA. Trans. R. Soc. Trop. Med. Hyg..

[19] Van Dam G.J., Stelma F.F., Gryseels B., Falcão Ferreira S.T.M., Talla I., Niang M., Rotmans J.P., Deelder A.M. (1996). Antibody response patterns against *Schistosoma mansoni* in a recently exposed community in Senegal. J. Infect. Dis..

[20] Ramanathan R., Burbelo P.D., Groot S., Iadarola M.J., Neva F.A., Nutman T.B. (2008). A Luciferase Immunoprecipitation Systems Assay Enhances the Sensitivity and Specificity of Diagnosis of *Strongyloides stercoralis* Infection. J. Infect. Dis..

[21] Norsyahida A., Riazi M., Sadjjadi S.M., Muhammad Hafiznur Y., Low H.C., Zeehaida M., Noordin R. (2013). Laboratory detection of strongyloidiasis: IgG, IgG 4 and IgE-ELISA s and cross-reactivity with lymphatic filariasis. Parasite Immunol..

[22] Lindo J.F., Lee M.G., Gillespie S., Pearson R.D. (2001). Strongyloides stercoralis and *S. fulleborni*. Principles and Practice of Clinical Parasitology.

[23] Bosqui L.R., Corral M.A., Levy D., Bydlowski S.P., Gryschek R.C.B., Custodio L.A., Pavanelli W.R., Conchon-Costa I., Costa-Cruz J.M., de Paula F.M. (2020). Evaluation of the Dot-ELISA as a diagnostic test for human strongyloidiasis based on the detection of IgA in saliva. Acta Trop..

[24] Bosqui L.R., Gonçalves A.L.R., Maria do Rosário F., Custodio L.A., de Menezes M.C.N.D., Murad V.A., de Paula F.M., Pavanelli W.R., Conchon-Costa I., Costa-Cruz J.M. (2015). Detection of parasite-specific IgG and IgA in paired serum and saliva samples for diagnosis of human strongyloidiasis in northern Paraná state, Brazil. Acta Trop..

[25] Babu S., Nutman T.B., Robert R., Thomas F., William S., Harry S., Anthony F., Cornelia W. (2019). Immune responses to helminth infection. Clinical Immunology.

[26] King C.L., Xianli J., Malhotra I., Liu S., Mahmoud A.A., Oettgen H.C. (1997). Mice with a targeted deletion of the IgE gene have increased worm burdens and reduced granulomatous inflammation following primary infection with *Schistosoma mansoni*. J. Immunol..

[27] Spencer L., Shultz L., Rajan T. (2001). V Interleukin-4 receptor–Stat6 signaling in murine infections with a tissue-dwelling nematode parasite. Infect. Immun..

[28] Gurish M.F., Bryce P.J., Tao H., Kisselgof A.B., Thornton E.M., Miller H.R., Friend D.S., Oettgen H.C. (2004). IgE enhances parasite clearance and regulates mast cell responses in mice infected with *Trichinella spiralis*. J. Immunol..

[29] Rihet P., Demeure C.E., Bourgois A., Prata A., Dessein A.J. (1991). Evidence for an association between human resistance to *Schistosoma mansoni* and high anti-larval IgE levels. Eur. J. Immunol..

[30] Faulkner H., Turner J., Kamgno J., Pion S.D., Boussinesq M., Bradley J.E. (2002). Age-and infection intensity-dependent cytokine and antibody production in human trichuriasis: The importance of IgE. J. Infect. Dis..

[31] Bethony J., Loukas A., Smout M., Brooker S., Mendez S., Plieskatt J., Goud G., Bottazzi M.E., Zhan B., Wang Y. (2005). Antibodies against a secreted protein from hookworm larvae reduce the intensity of hookworm infection in humans and vaccinated laboratory animals. FASEB J..

[32] De Moira A.P., Fulford A.J.C., Kabatereine N.B., Ouma J.H., Booth M., Dunne D.W. (2010). Analysis of complex patterns of human exposure and immunity to *Schistosomiasis mansoni*: The influence of age, sex, ethnicity and IgE. PLoS Negl. Trop. Dis..

[33] Hirata T., Uchima N., Kishimoto K., Zaha O., Kinjo N., Hokama A., Sakugawa H., Kinjo F., Fujita J. (2006). Impairment of host immune response against *Strongyloides stercoralis* by human T cell lymphotropic virus type 1 infection. Am. J. Trop. Med. Hyg..

[34] Matowicka-karna J., Kemona H. (2014). IgE antibodies in toxoplasmosis Przeciwciała IgE w toksoplazmozie. Postepy Hig. Med. Dosw. Online.

[35] Lindo J.F., Atkins N.S., Lee M.G., Robinson R.D., Bundy D.A.P. (1996). long-term serum antibody isotype responses to *Strongyloides stercoralis* filariform antigens in eight patients treated with ivermectin. Am. J. Trop. Med. Hyg..

[36] Rodrigues R.M., Sopelete M.C., De Oliveira Silva D.A., Cunha-Júnior J.P., Taketomi E.A., Costa-Cruz J.M. (2004). *Strongyloides ratti* antigenic components recognized by IgE antibodies in immunoblotting as an additional tool for improving the immunodiagnosis in human strongyloidiasis. Mem. Inst. Oswaldo Cruz.

[37] De Jesus Inês E., Silva M.L.S., Souza J.N., Teixeira M.C.A., Soares N.M. (2013). The role of glycosylated epitopes in the serodiagnosis of *Strongyloides stercoralis* infection. Diagn. Microbiol. Infect. Dis..

[38] Ahmad H., Balachandra D., Arifin N., Nolan T.J., Lok J.B., Hayat Khan A., Yunus M.H., Noordin R. (2020). Diagnostic Potential of an IgE-ELISA in Detecting Strongyloidiasis. Am. J. Trop. Med. Hyg..

[39] Yunus M.H., Arifin N., Balachandra D., Anuar N.S., Noordin R. (2019). Lateral Flow Dipstick Test for Serodiagnosis of Strongyloidiasis. Am. J. Trop. Med. Hyg..

[40] Arifin N., Yunus M.H., Nolan T.J., Lok J.B., Noordin R. (2018). Identification and Preliminary Evaluation of a Novel Recombinant Protein for Serodiagnosis of Strongyloidiasis. Am. J. Trop. Med. Hyg..

[41] Sambrook J., Russell D. (2000). Molecular Cloning: A Laboratory Manual.

[42] Krolewiecki A., Nutman T.B. (2019). Strongyloidiasis: A Neglected Tropical Disease. Infect. Dis. Clin. N. Am..

[43] Kennedy M.B. (1995). Origin of Pdz (Dhr, Glgf) domains. Trends Biochem. Sci..

[44] Harris B.Z., Lim W.A. (2001). Mechanism and role of PDZ domains in signaling complex assembly. J. Cell Sci..

[45] Niethammer M., Kim E., Sheng M. (1996). Interaction between the C terminus of NMDA receptor subunits and multiple members of the PSD-95 family of membrane-associated guanylate kinases. J. Neurosci..

[46] Dev K.K. (2004). Making protein interactions druggable: Targeting PDZ domains. Nat. Rev. Drug Discov..

[47] Mu Y., Huang H., Liu S., Cai P., Gao Y. (2012). Molecular characterization and ligand binding specificity of the PDZ domain-containing protein GIPC3 from *Schistosoma japonicum*. Parasit. Vectors.

[48] Rodrigues R.M., de Oliveira M.C., Sopelete M.C., Silva D.A.O., Campos D.M.B., Taketomi E.A., Costa-Cruz J.M. (2007). IgG1, IgG4, and IgE antibody responses in human strongyloidiasis by ELISA using *Strongyloides ratti* saline extract as heterologous antigen. Parasitol. Res..

[49] Varatharajalu R., Parandaman V., Ndao M., Andersen J.F., Neva F.A. (2011). *Strongyloides stercoralis* excretory/secretory protein strongylastacin specifically recognized by IgE antibodies in infected human sera. Microbiol. Immunol..

[50] Ramachandran S., Thompson R.W., Gam A.A., Neva F.A. (1998). Recombinant cDNA clones for immunodiagnosis of strongyloidiasis. J. Infect. Dis..

