# Supplementary material for: Strongyloides-Specific IgE Phage cDNA Clones and Development of a Novel ELISA for Strongyloidiasis
Source: Diagnostics (Basel). 2021 May 28;11(6):985. doi: 10.3390/diagnostics11060985 (PMC8228214; doi:10.3390/diagnostics11060985)
Supplement: Supplementary file 1 [file diagnostics-11-00985-s001.zip › diagnostics-1249182 supp.pdf]

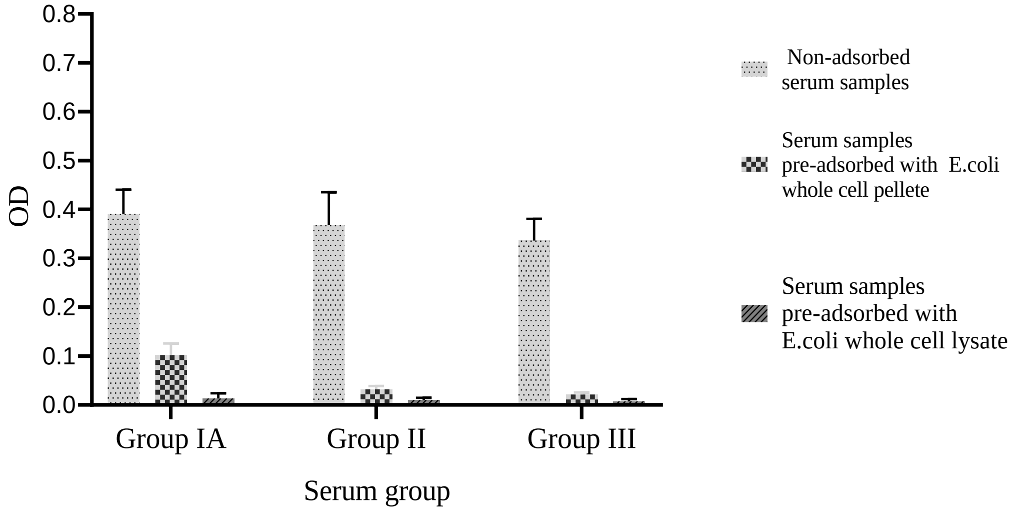

Figure S1. OD_{405} readings of Group IA, Group II, and Group III serum samples before pre-adsorption and after pre-adsorption processes with whole-cell and lysate of *E. coli.*

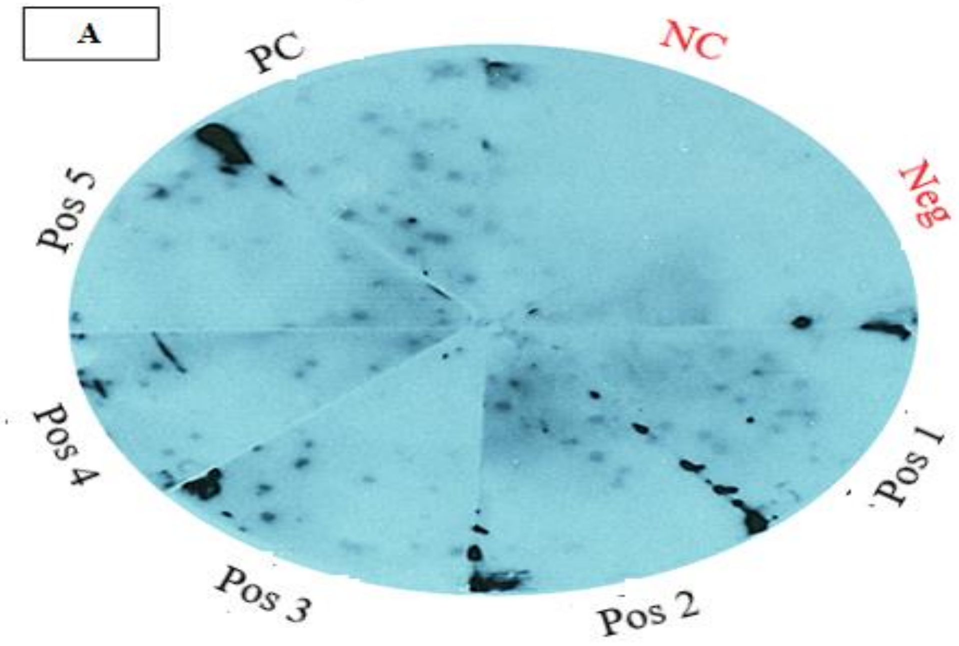

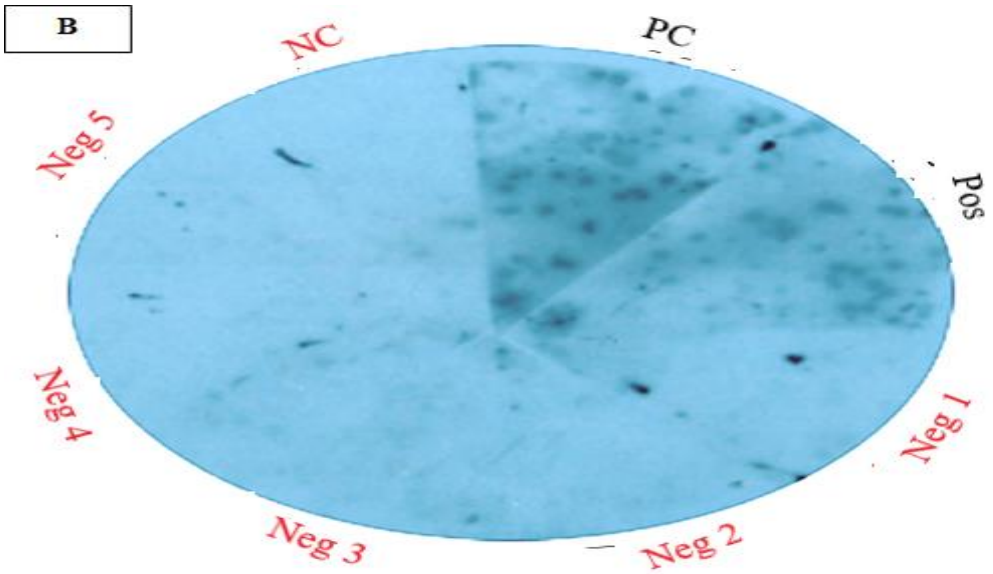

Figure S2. A. Reactivity with positive sera **B**. non-reactivity with negative sera of clone A133 using pre-adsorbed individual serum samples and probed with IgE -HRP. Each triangular NC membrane section was screened with different positive and negative serum samples. Reactive clones (dark spots) represents positive results and non-reactive clones represent negative results. PC-pooled positive control; NC-pooled negative control; Pos- individual positive serum; Neg-individual negative serum.
